# Characteristics and outcomes of emergency department patients across health care systems: an international multicenter cohort study

**DOI:** 10.1186/s12245-024-00715-0

**Published:** 2024-09-27

**Authors:** Bas de Groot, Nicoline T. C. Meijs, Michelle Moscova, Wouter Raven, Menno I. Gaakeer, Wendy A. M. H. Thijssen, Heleen Lameijer, Amith Shetty, Annmarie T. Lassen

**Affiliations:** 1 Department of Emergency Medicine, Radboud University Medical Centre, Postbus 9101, 6500 HB, Geert Grooteplein Zuid 22, Nijmegen, the Netherlands; 2 Research Centre for Emergency Medicine, Aarhus University Hospital, Aarhus, Denmark; 3 Department of Emergency Medicine, Leiden University Medical Centre, Leiden, the Netherlands; 4 Faculty of Medicine and Health, University of New South Wales, Sydney, Australia; 5 Department of Emergency Medicine, ADRZ Hospital, Goes, The Netherlands; 6 Department of Emergency Medicine, Catharina Hospital, Eindhoven, The Netherlands; 7 Department of Emergency Medicine, Medical Center Leeuwarden, Leeuwarden, the Netherlands; 8 Biomedical Informatics and Digital Health, University of Sydney, New South Wales, Australia; 9 Department of Emergency Medicine, Odense University, Odense, Denmark

**Keywords:** Emergency department, Health-care systems, Quality of care, Template for uniform reporting

## Abstract

**Background:**

A wide variation of emergency medical system configurations across countries has limited the value of comparison of quality and performance measures in the past. Furthermore, lack of quantitative data on EDs prevents definition of the problems and possibilities for data driven improvement of quality of care. Therefore, the objective is to describe and compare Emergency Department (ED) populations and characteristics, and their outcomes in the Netherlands, Denmark and Australia, using a recently developed template for uniform reporting of standardized measuring and describing of care provided in the ED (structure, staffing and governance, population, process times and outcomes).

**Methods:**

This international multicenter cohort included all consecutive ED visits from National Quality Registries or Databases from participating sites from three countries. Patient and ED characteristics (using the template for uniform reporting) and relevant clinical outcomes were described and compared per country.

**Results:**

We included 212,515 ED visits in the Netherlands, 408,673 in Denmark and 556,652 in Australia. Patient characteristics differed markedly, with Australian ED patients being younger, less often triaged as “immediate”, and less often triaged with the high-risk chief complaints “feeling unwell” compared to Danish and Dutch patients. ED characteristics mainly differed with respect to the mean annual census per ED (Netherlands 26,738 (SD 2630), Denmark 36,675 (SD 12974), Australia 50,712 (4884)), median (IQR) lengths of stay of patients discharged home (Netherlands 2.1 (1.4–3.1); Denmark 2.8 (1.7–5.0); Australia 3.3 (2.0–5.0) hrs) and proportion of hospitalizations (ranging from 30.6 to 39.8%).

In-hospital mortality was 4.0% in Australia, higher compared to the Netherlands and Denmark (both 1.6%). Not all indicators of the framework were available in all registries.

**Conclusions:**

Patient and ED characteristics and outcomes varied largely across countries. Meaningful interpretation of outcome differences across countries could be improved if quality registries would more consistently register the measures of the recently developed template for uniform reporting.

**Supplementary Information:**

The online version contains supplementary material available at 10.1186/s12245-024-00715-0.

## Introduction

Quality of care is important, yet poorly defined for the emergency department (ED) setting, despite a vast amount of research into quality indicators [1–3]. Importantly, a wide variation of emergency medical system configurations across countries has limited the value of comparison of quality and performance measures in the past [4]. Furthermore, lack of quantitative data on EDs prevents definition of the problems and possibilities for data driven improvement of quality of care [5]. This is a concern because quality of ED care is continuously threatened by overcrowding with more complex older patients, shortage of medical and nursing staff and dynamic decision processes based on limited information on life-threatened patients [6, 7].

An international group of experts developed a template for uniform reporting of standardized measuring and describing of care provided in the ED [4]. The final measures and their definitions were 1) Structure, 2) Staffing and governance, 3) Population, 4) Process times, 5) Hospital and healthcare system, and (6) Outcomes. Although this template was applied on a national level [6, 8], it has not yet been used to compare ED systems across countries.

Currently, prehospital and ED care is organized differently across health-care systems [9–11]. For example, the number of general practitioners (GPs) and EDs per 100,000 inhabitants differs considerably across countries [11], and Australian EDs are run by emergency physicians, while in the Netherlands emergency physicians work together with other specialties. These differences may be justified to some extent because organization of ED care is partially determined or limited by political, cultural, financial and geographical differences. Nevertheless, organization of emergency medical services is often not evidence-based, potentially affecting outcomes. For example, reduction of the number of EDs in a health-care system can result in longer transport times to the ED, leading to higher initial disease severity of patients upon arrival in the ED, potentially affecting hospitalization and mortality. Conversely, a reduction in EDs will increase patient volumes into the remaining EDs, which in turn increases exposure of medical personnel. This may have a positive effect (practise makes perfect), or a negative effect (ED overcrowding).

Quality registries could be used to compare ED cohorts characteristics and relevant clinical outcomes using this template across countries. It would be an important first step to assess which data of this template are available in these registries. If such data are available and differences in relevant clinical outcomes exist, in-depth analyses of these differences could help to provide a more evidence-based approach to optimize emergency medical systems, strongly promoted in the World Health Assembly Resolution 60.22 [12].

The aim of this study was therefore to describe and compare ED cohorts, characteristics and their outcomes in three different health-care systems (Netherlands, Denmark and Australia), using the set of measures of a previously developed template for uniform reporting (structure, staffing and governance, population, process times and outcomes).

## Methods

### Study design

An international multicenter cohort study, using data from three quality registries from participating sites in the Netherlands, Denmark and Australia.

### Study setting

For a detailed description of the study settings see supplementary file 1 and references [9, 13–16] for the Netherlands, [10, 17, 18] for Denmark and [19–22] for Australia.

The medical ethics review committee of Leiden Den Haag Delft (METC LDD)) declared that the research did not fall under the Medical Research Act, and waived the need for informed consent since this was an observational study (file no. G21.204). Low to Negligible risk ethics approval was granted for the study and use of data from the Western Sydney Local health District ethics committee.

### Study population

All consecutive ED patients were included. See for inclusion dates supplementary file 2.

### Data collection

Data were collected from three databases. Uniformity of variable definitions were discussed and checked by the researchers of the three participating countries.

#### Netherlands

The Netherlands Emergency department Evaluation Database (NEED) contained information of four hospitals between the period 1 January 2017 until 1 September 2021: one tertiary care center and three small to large urban hospitals.

#### Denmark

The Danish database contained information of all ED visits from patients above 18 years old in five different EDs (four individual hospitals) from 1 January 2016 until 19 March 2018.

#### Australia

The Australian database contained information of all ED visits in three hospitals and data of all adult ED patients (> 16 years) in one hospital from January 2017 to November 2019.

For a detailed description of data collection, see supplementary file 2 and 3 and reference [23]. Briefly, we collected demographic data, arrival by ambulance, involved specialties, triage categories and presenting complaints, vital signs, diagnostic tests. The top 18 presenting complaints were merged among the different triage systems (see supplementary file 4).

Sample size calculations can be found in supplemental file 5.

For exact variable definitions of data collected and shown in Tables 1, 2 and 3 see supplemental files 6 to 8.

**Table 1:** Patient characteristics per country

	**Netherlands**	**Denmark**	**Australia**
**Demographics**			
N (%)	212,515 (100)	408,673 (100)	556,652 (100)
Age, median (IQR)	55 (29–72)	55 (35–73)	39 (24–61)
Male Sex N(%)	109,158 (51.4) [0.2]	205,784 (50.4) [0.5]	280,366 (50.4)
**Urgency**			
Triage system present	MTS/NTS	DEPT	ATS
Triage category N(%)	[3.9]	[19.2]	
Non-urgent & standard	68,085 (32.1)	178,261 (43.6)^#^	205,398 (36.9)*
Urgent	87,039 (41.0)	91,857 (22.5)^#^	198,850 (35.7)*
Very urgent	42,483 (20.0)	51,948 (12.7)^#^	143,935 (25.9)*
Immediate	6484 (3.1)	8028 (2.0)^#^	8469 (1.5)*
**Top 18 presenting complaints, N (%)**	[4.6]	[14.3]	[0.5]
Extremity problems	45,645 (21.5)	9125 (2.2)^#^	24,865 (4.5)*
Feeling unwell	31,370 (14.8)	62,779 (15.4)^#^	41,327 (7.4)*
Abdominal pain	21,836 (10.3)	37,683 (9.2)	58,690 (10.6)
Dyspnea	17,823 (8.4)	24,240 (5.9)^#^	25,140 (4.5)*
Chest pain	16,063 (7.6)	14,879 (3.6)#	48,055 (8.7)
Trauma	13,088 (6.2)	1472 (0.4)^#^	85,264 (15.3)*
Minor trauma	-	143,687 (35.2)^#^	-
Wounds	9435 (4.4)	419 (0.1)^#^	-
Collapse	5121 (2.4)	4807 (1.2)^#^	9200 (1.7)*
Palpitations	4957 (2.3)	3461 (0.8)^#^	-
Urinary problems	4026 (1.9)	-	-
Headache	3686 (1.7)	1546 (0.4)^#^	12,681 (2.3)*
Overdose & poisoning	2895 (1.4)	5007 (1.2)	-
Facial problems	2586 (1.2)	-	-
Diarrhea & vomiting	2582 (1.2)	1891 (0.5)^#^	19,649 (3.6)*
Eye problems	2403 (1.1)	-	11,585 (2.1)*
Abscesses & local infections	2400 (1.1)	3229 (0.8)	10,858 (2.0)
Behaving strangely or suicidal	2133 (1.0)	-	-
Back pain	1953 (0.9)	2545 (0.6)^#^	9565 (1.7)*
Care – patient review	-	-	22,087 (4.0)
Mental health	-	-	18,431 (3.3)
Coughing	-	-	8940 (1.6)
Bleed per vaginum	-	-	8764 (1.6)
LAB abnormalities	-	7156 (1.8)	-
GI bleeding	-	4592 (1.1)	-
Other	12,779 (6.0)	21,757 (5.3)^#^	138,707 (24.9)*
**Co-morbidity**			
Charlson Comorbidity Index Median (IQR)	-	0 (0–1) [3.0]	-
Specialty (first contact), N(%)	[5.7]	[2.9]	-
Emergency physician^*^	33,910 (16.0)	44,261 (10.8)	
Medicine ^a^	62,576 (29.4)	157,440 (38.5)	
Surgery ^b^	48,182 (22.7)	179,404 (43.9)	
Pediatrics	6854 (3.2)	48 (0.0)	
Others	48,814 (23.0)	15,600 (3.8)	
**Disease severity**			
Arrival by ambulance N (%)	60,377 (28.4) [6.9]	-	163,108 (28.1)*
**If vital signs are registered** N(%)^c^			
No vital signs measured	72,784 (34.2)	252,649 (61.8)^#^	75,703 (13.60)*
One or some vital signs measured	63,984 (30.1)	49,750 (12.2)^#^	295,890 (53.2)*
All vital signs measured	75,747 (35.6)	106,274 (26.0)^#^	185,069 (33.2)*
Mean (SD) or Median (IQR)			
O_2_ saturation by peripheral pulse oximetry	97.1 (5.41) [44.8]	96.6 (4.8) [36.7]	98 (1.99) [15.2]
Respiratory rate (/min)	18 (7.36) [58.1]	18.4 (5.1) [35.2]	21(6.90) [18.1]
Systolic RR (mmHg)	139 (30.94) [49.6]	137 (24.1) [37.6]	135(22.42) [30.6]
Diastolic RR (mmHg)	83 (16.54) [49.7]	78 (15.7) [37.6]	78 (12.51) [30.7]
Heart rate (/min) (by pulse oximetry or monitor leads)	84 (72–99) [53.9]	83 (72–97) [37.7]	88 (76–102) [16.8]
GCS N(%)			
Not registered	187,479 (88.2)	305,884 (74.8)	326,851 (58.7)*
GCS = 15	23,116 (10.9)	93,225 (22.8)^#^	212,696 (38.2)*
GCS < 15	1920 (0.9)	9564 (2.3)^#^	17,105 (3.1)*
Body temperature (^o^C) measured by tympanic or rectal meter	36.9 (1.74) [45.0]	37.0 (0.9) [29]	36.8 (1.2) [29.2]
**Diagnostic tests**			
Blood tests (Yes) N(%)	122,547 (57.7) [0.1]	231,075 (56.5)^#^	331,922 (59.6)*
**If blood test is registered** Median (IQR)			
Thrombocytes	244 (196–302) [6.9]	249 (201–307)[49.6]	243 (195–299) [1.9]
Creatinine (µmol/L)	76 (63–96) [45.4]	78 (64–97) [55.3]	72 (58–93) [1.7]
Bilirubin (mmol/L)	11 (7–16) [89.7]	8 (6–12) [42.1]	8 (6–13) [14.5]
Lactate (mmol/L)	1.5 (1.1–2.3) [89.9]	1.2 (0.8–1.9) [17.1]	1.5 (1.1–2.1) [3.5]
Blood cultures taken (Yes) N(%)	16,469 (7.7) [30.3]	56,546 (13.8)^#^	43,040 (7.7)*
Urine culture (Yes) N(%)	9920 (4.7) [30.4]	46,199 (11.3)^#^	51,741 (9.3)*
Radiological imaging ^d^ (Yes) N(%)	120,542 (56.7) [0.1]	122,850 (30.1)^#^	-

Patient characteristics are presented for three different countries. Normally distributed data is presented as mean (SD), skewed data as median (IQR) and categorical data as number (%)

*Abbreviations*: *SD* standard deviation, *IQR* interquartile range, *N* number, *GCS* Glascow Coma Scale, *ED* emergency department, *MTS* Manchester Triage System, *NTS* Netherlands Triage System, *DEPT* Danish Emergency Process Triage, *ATS* Australian Triage System

For a detailed description of the variables see supplementary file 6

^*^P < 0.001 Australia compared to the Netherlands

^#^*P* < 0.001 Denmark compared to the Netherlands

‘ – ‘ = not available,. “[.]” = percentage of missing data between square brackets, when the percentage was 0.0 this was not noted

^#^In the Danish database all ED visits in the "minor trauma " part of the ED are registered in this category—and not in the extremity category. Most patients in the minor trauma catrgoy had minor injuries, i.e. a twisted ankle

^*^Many patients registered for a certain specialty are seen by the emergency physician but registered for a specialty

^a^Medicine contains internal medicine, pulmonary medicine, cardiology, neurology

^b^Surgery contains general surgery, trauma surgery, orthopedic surgery, urology, ENT (ear, nose, throat), ophthalmology

^c^These are the initial vital signs before ED treatment

^d^Radiological imaging is positive when an X-ray, an ultrasound and/or a CT scan was performed

**Table 2:** ED, hospital, healthcare and population characteristics according to Utstein framework per country

	**Netherlands**	**Denmark**	**Australia**
**Variable**			
**Health care system**			
*Number of GPs / 1000 inhabitants*	0.74	0.65	1.21
*Number of ambulance staffing / 1000 inhabitants*	0.38	0.58	0.38
*Target time to patient*	Within 15 min for highest priority	Within 15 min for highest priority	Within 15min for emergency cases and 10min for highest priority
**Type of hospital**			
*Number of Acute Care beds / 1000 inhabitants*	2.62	2.47	-
*Number of hospital beds / 1000 inhabitants*	2.3 = national 1.83 = database	2.50	2.70 = regional
Number of hospitals in registry / database	4	5	4
Number of ED visits in registry / database	212,515	408,673	556,652
Number of persons in Adherence areas of hospitals included in registry / database	797,388	1,221,000	946,000
**ED structure**			
Number of treatment spaces	89	80*	-
Number of resuscitation spaces	13	10*	-
Number of short stay unit spaces	-	0*	-
**Governance / staffing**			
Number of direct clinical care hours by physicians per 100 ED visits	168	122*	-
Number of direct clinical care hours by nurses per 100 ED visits	-	229*	-
Emergency medicine specialist in the ED 24/7	Yes 3 of 4 EDs in database	No*	Yes
**Population**			
*Total ED census (N)*			-
Number of ED visits/year per ED in database. Mean (SD)	26,738 (2630)	37,724	50,712 (4884)
Infant / pediatric population 0–5 years N(%)	10,644 (5.0)	0	44,938 (8.1)
Geriatric population (> 75 years) N(%)	39,924 (18.9)	85,422 (20.9)	64,340 (11.6)
Number of patients arriving by ground or air ambulance (%)	60,377 (28.4) [6.9]		163,108 (28.1)
Number of patients with high acuity level ^a^	6484 (3.1)	8028 (2.0)	8469 (1.5)
Number of patients with low acuity level ^b^	68,085 (32.1)	178,261 (43.6)	205,398 (36.9)
**Process times**			
Lengths of ED stay (hours) of all ED patients Median (IQR)	2.4 (1.5–3.5)	2.9 (1.7–5.2) [0.4]	-
Lengths of ED stay (hours) of ED patients discharged home Median (IQR)	2.1 (1.4–3.1)	2.8 (1.7–5.0)	3.3 (2.0–5.0)
**Outcomes**			
Disposition, N(%)	[5.6]		
Home	91,460 (43.0)	283,463 (69.4)	361,814 (65.0)
Normal ward or CDU	75,561 (35.6)	118,799 (29.1)	95,874 (17.2)
Transfer to other hospital	3448 (1.6)	3879 (0.9)	478 (0.1)
MCU or CCU	3535 (1.7)	-	
ICU or HDU	2071 (1.0)	2532 (0.6)	3734 (0.7)
Short stay unit	-	-	73,671 (13.2)
7-day ED revisit, N(%)	10,263 (4.8) [0.1]	31,620 (7.7)	-

ED, hospital, health care and population characteristics are presented as follows: Normally distributed data is presented as mean (SD), skewed data as median (IQR) and categorical data as number (%). For a detailed description of the variables see supplementary file 6. The text in *italics* is national data obtained from the literature

*Abbreviations*: *GP* general practitioner, *ED* emergency department, *SD* standard deviation, *IQR* interquartile range, *N* number, *MTS* Manchester Triage System. *CDU* clinical decision unit, *MCU* medium care unit, *CCU* coronary care unit, *ICU* intensive care unit, *HDU* high dependency unit, ‘ – ‘ = not available,. “[.]” = percentage of missings between square brackets, when the percentage was 0.0 this was not noted. For a detailed description of the variables and the sources used for the data see supplementary file 7

^a^High acuity level includes very urgent, immediate care required, i.e. shock, coma, advanced life support

^b^Low acuity level includes low urgency, i.e. twisted ankle, wound, contusion, insect bite

^*^Based on one ED

**Table 3:** Relevant clinical outcomes per country

	**Netherlands**	**Denmark**	**Australia**
**Lengths of ED stay** (hours) for all ED patients. Median (IQR)	2.4 (1.5–3.5)	2.9 (1.7–5.2) [0.4]	-
**Length of ED stay** (hours) for ED patients discharged home Median (IQR)	2.1 (1.4–3.1)	2.8 (1.7–5.0)	3.3 (2.0–5.0)
**Hospital admission** N(%) directly after ED stay			
Number	84,615	125,210	188,324
% (95-confidence interval)	39.8 (39.6–40.0)	30.6 (30.3–30.7)	33.8 (33.7–33.9)*
Geriatric population (> 75 years) % (95-confidence interval)	61.7 (61.5–61.9)	-	70.1 (70.0–70.2)*
Infant population (0–5 years) % (95-confidence interval)	29.3 (29.1–29.5)	-	11.4 (11.3–11.5)*
**Disposition**, N(%)	[5.6]		
Home^a^	115.899 (54.5)	283,463 (69.4)^#^	361,814 (65.0)*
Normal ward or CDU	75,561 (35.6)	118,799 (29.1)^#^	95,874 (17.2)*
Transfer to other hospital	3448 (1.6)	3879 (0.9)^#^	478 (0.1)*
MCU or CCU	3535 (1.7)	-	-
ICU or HDU	2071 (1.0)	2532 (0.6)^#^	3734 (0.7)*
Short stay unit	-	-	73,671 (13.2)
**Lengths of hospital stay** (days) of hospitalized ED patients Median (IQR)	3.0 (1.0–6.0)	3.0 (1.0–6.0)	1.8 (0.5–4.8)
**In-hospital mortality** N (%)			
Total cohort	3497 [1.1]	6879	22,390
% (95-confidence interval)	1.6 (1.54–1.65)	1.6 (1.56–1.64)	4.0 (3.94–4.05)*
Geriatric population (> 75 years) % (95-confidence interval)	4.5 (4.41–4.59)	-	18.3 (18.20–18.40)*
Infant population (0–5 years) % (95-confidence interval)	0.04 (0.031–0.049)	-	0.03 (0.025–0.035)
**30-day mortality** N (%)	-	13,721 (3.4) [0.5]	-
**7-day ED revisit**, N(%)	10,263 (4.8) [0.1]	31,620 (7.7)^#^	-

Outcomes are presented as follows: Normally distributed data is presented as mean (SD), skewed data as median (IQR) and categorical data as number (%). ‘ – ‘ = not available. “[.]” = % missing data

*Abbreviations*: *ED* emergency department, *IQR* interquartile range, *N* number, *CDU* clinical decision unit, *MCU* median care unit, *CCU* coronary care unit, *ICU* intensive care unit, *HDU* high dependency unit. For a detailed description of the variables see supplementary file 8

^*^P < 0.001 Australia compared to the Netherlands

^#^*P* < 0.001 Denmark compared to the Netherlands

^a^Including patients who were discharged home and were follow-up at an outpatient department

### Outcome measures

In-hospital mortality (including death on arrival or in the ED) and hospitalization (to any ward or unit including transfers to another hospital) were the primary outcomes. Secondary outcomes were ED and hospital length of stay, and number of ED revisits.

### Statistical analyses

Data were presented as mean with standard deviation (SD) when normally distributed. Skewed data were presented as median with interquartile range (IQR). Categorical data were presented as number with percentages.

Differences among countries could only be calculated using aggregated data because data of individual patients were not allowed to cross borders. We used an online calculator to calculate differences of N (%) in Tables 1 and 3 (www.evanmiller.org/ab-testing/chi-squared.html). Outcome measures like in-hospital mortality were reported with 95% confidence intervals (95%-CI).

If 95%-confidence intervals did not overlap differences were considered to be significant. We adjusted the P value for multiple testing according to the method of Bonferroni. A P-value < 0.05 was considered to be statistically significant.

Data were analyzed using SPSS (SPSS, version 25.0, IBM, New York, USA) in the Netherlands and SAS (version 9.4) in Australia. In Denmark, STATA (version 16) was used to analyze the Danish data.

#### Handling of missing data

The percentage of missing data were provided in between square brackets in the tables and provided insight in the feasibility of fair comparison of countries. Missing data could be data which were not registered, i.e. vital signs are often not measured in patients with a twisted ankle. Impossible (based on expert opinion) or missing values were set to missing.

## Results

### Patient flow through the study

**Fig. 1:**
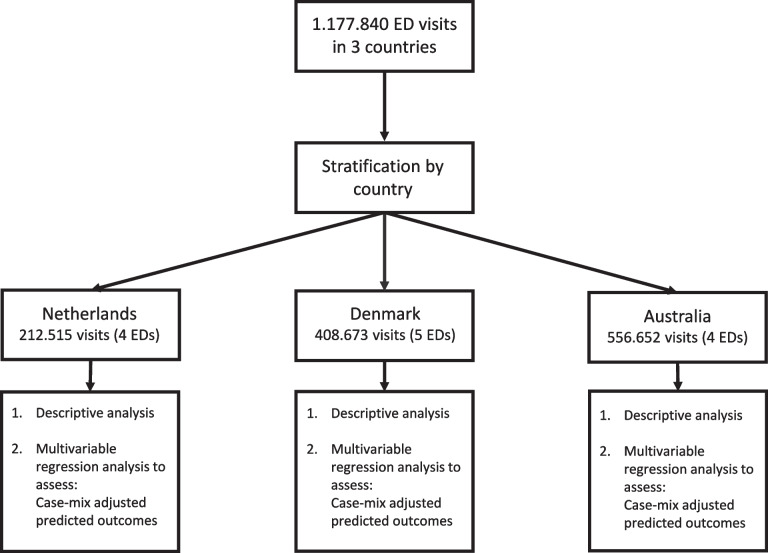
Patient flow through study

Figure 1 shows patient flow through the study. In total, 1,177,840 ED visits in three countries were included; 212,515 patients in the Netherlands, 408,673 patients in Denmark and 556,652 patients in Australia.

### Patient characteristics per country

Table 1 shows patient characteristics for each country. The median age varied widely among countries, being 39 (24–61) in Australia compared to 55 (25–73) in Denmark and 55 (29–72) in the Netherlands. The proportion of male patients was approximately 51%, comparable in the three countries. The proportion of patients triaged with the highest acuity level was lower in Australia (1.5%) compared to the Netherlands (3.1%) and different triage systems were used. In the Netherlands and Denmark mean oxygen saturation tended to be lower with a higher standard deviation compared to the Australian ED patients. However, the percentage of patients with a GCS lower than 15 was highest in Australia (3.1%) compared to 2.3% in Denmark and 0.9% in the Netherlands. The percentage of patients in whom no vital signs were measured was much lower in Australia (13.6%) compared to Denmark (61.2) and the Netherlands (34.2%). The percentage of patients who arrived by ambulance was similar in the Netherlands (28.4%) and Australia (28.1%).

The top 18 most common presenting complaints was similar across countries but their frequency differed with respect to several presenting complaints. For example, compared to Denmark and the Netherlands, the previously reported high-risk presenting complaint “feeling unwell”(23) occurred almost 50% less often in Australia. In the Netherlands, 8.4% had “dyspnea”, which was higher than in Denmark (5.9%) or Australia (4.5%). The proportions of “Chest pain” in the Netherlands (7.6%) and Australia (8.7%) were more than double of that of Denmark (3.6%). In Denmark, only 0.1% of the patients had the presenting complaint "collapse" compared to 2.4% in the Netherlands and 1.7% in Australia. Blood tests values were comparable across countries, but blood cultures were more often taken in Denmark (13.2%) compared to the Netherlands and Australia (both 7.7%). In the Netherlands radiological imaging was more often performed compared to Denmark (56.7% vs 30.1%).

### ED characteristics per country

Table 2 shows the ED, hospital and health care characteristics per country. The number of GPs per 1000 inhabitants was higher in Australia (1.21) compared to the Netherlands (0.74), while the number of ambulance staffing per 1000 inhabitants was 0.38, similar in Australia and the Netherlands. The annual number of ED visits was higher in Australia (50.711 visits annually) compared to the Netherlands (26.738 visits annually). The percentage of geriatric patients was 11.6% in Australia, lower than in the Netherlands (18.9%). Finally, in Australia there was 24/7 staffing of the ED which was not the case in the Netherlands and Denmark.

### Relevant clinical outcomes per country

ED length of stay for patients discharged home in the Netherlands was 2.1 h, much lower than the 3.3 h in Australia (Table 3).

In Table 3 it is also shown that the proportion of hospitalized ED patients was 33.8% in Australia and 30.6% in Denmark, lower than the 39.8% in the Netherlands. In Australian children aged 0–5 years 11.4 (11.3–11.5)% were hospitalized, compared to 29.3 (29.1–29.5) in the Netherlands.

The median hospital lengths of stay was 3 days in the Netherlands and Denmark, longer than the 1.8 days in Australia. In-hospital mortality was 4.0% in Australia, compared to 1.6% in Denmark and the Netherlands. In the geriatric population in-hospital mortality was more than four times higher in Australia (19.3%) compared to the Netherlands (4.5%). Finally, the number of 7-day ED revisits was 7.7% in Denmark, higher than the 4.8% in the Netherlands.

## Discussion

Patient and ED characteristics and outcomes varied largely across countries, with a large variation of in-hospital mortality. Meaningful interpretation of these different outcomes is only possible if the measures proposed in the recently developed template for uniform reporting would be more consistently registered.

Studies investigating differences in patient, ED-characteristics and outcomes, using the recently developed template for uniform reporting according to the Utstein-style guidelines (4), are scarce. Two studies using the template on a national level showed that ED structure and staffing between EDs within one country differed considerably [6, 8]. We showed that ED systems across countries also varied largely. In addition, several studies comparing healthcare systems of different countries exist [11]. Baier et al. showed that large differences among countries exist in the organization of prehospital and ED care and kind of payment systems but the lack of use of a uniform template as used in the present study complicates direct comparisons of outcomes (11).

In the present study we attempted to compare countries in a more uniform way so that factors can be identified which could potentially be used to improve outcomes. However, not all measures of the template for uniform reporting were consistently registered in the quality registries. In addition, fair comparison of outcomes across countries requires appropriate case-mix adjustments. Because legislation does not allow data of individual patients to cross borders, multivariable regression cannot be used for such case-mix corrections. In the future case-mix adjustments maybe possible with federated learning techniques [24]. Development of appropriate models for case-mix adjustments with federated learning would probably require important variables like co-morbidity, frailty and obesity, which are currently lacking in the registries.

### Patient characteristics at ED presentation

Nevertheless, some of the differences in patient characteristics at ED presentation are worthwhile to discuss because these characteristics provide information about the pre-hospital setting. Compared to Dutch and Danish patients, Australian ED patients were younger, were more often self-referrals (75% see supplemental file 1 compared to ~ 33% in the Netherlands, see www.stichting-need.nl), had a lower proportion of the highest acuity, and less often high-risk presenting complaints like “feeling unwell”(23). In addition, the proportion of patients discharged home from the ED was approximately 50% higher in Australia. The large proportion of self-referrals and apparent lower disease severity of the Australian population may be explained by the different financing and payment of pre-hospital and emergency care, while in the Netherlands and Denmark the GP may function more as a gatekeeper for the hospitals preventing many unnecessary ED visits. The observation that in the Netherlands and Denmark more patients present with the highest acuity level is more difficult to explain. On the one hand, it could be hypothesized that survivor bias plays a role in Australia where larger distances to hospitals may limit access to EDs explaining the lower proportion of patients with the highest acuity, but it is also possible that Danish and Dutch ED patients recognize their disease severity later and subsequently present to the ED sicker compared to Australian ED patients. It is also possible that the combination of keeping away unnecessary ED visits and not timely sending in sick patients by GPs might be an explanation for the higher acuity level in the Netherlands and Denmark. This could be a downside of the Dutch system in which GPs are gatekeepers of the hospitals. Only in-depths analyses could clarify these differences and identify opportunities for improvement in prehospital emergency care.

### ED characteristics

In Australia, the annual census of ED visits and thus input of patients is two times higher compared to the European countries (Table 2), which could explain the longer through-put times as reflected in the longer ED length of stay of patients discharged home in Australia. Output of patients, i.e. exit blocks, are likely to be similar since the number of beds of 2.7 per 1000 inhabitants is slightly higher in Australia compared to Netherlands and Denmark (Table 2). If workload and the risk of ED overcrowding is higher in Australia compared to Denmark and the Netherlands, this may contribute to the differences in mortality [25], and may also contribute to the lower proportion of patients who are hospitalized compared to the Netherlands.

### Relevant clinical outcomes

In-hospital mortality of ED patients in Australia is much higher than in the two European countries, especially in the geriatric population. Although it is possible that older patients in Australia are more frail or have more co-morbidity than in the Netherlands, it is likely that the selection of older patients visiting the ED is different in Australia. For example, in Australia it is less common for older people to die at home, instead people go to a hospital sooner, also if they have a DNR code. This information is currently lacking in the quality registries but is extremely important to take into account if meaningful conclusions about quality of care of the emergency medical services are to be taken.

In 2019 the Netherlands spent 10.17% of the Gross Domestic Product (GDP) on healthcare, which is slightly more compared to the 9.96% in Denmark and 9.42% in Australia [26], which may be partially explained by the markedly longer median length of hospital stay in the Netherlands compared to Australia. It is possible that the Dutch patients are admitted unnecessarily long compared to Australia. In the future, it could be examined whether this is indeed the case and whether healthcare costs can be reduced by shortening hospital length of stay. Interestingly, the annual census per ED is higher in Denmark compared to the Netherlands while in-hospital mortality is the same, suggesting that reduction of EDs with the argument that “practice makes perfect” is not necessarily applicable to the Dutch and Danish setting as has been suggested in a recent Danish study, despite the fact that this is recently suggested in policy statements in the Netherlands [27, 28].

The template was developed to function as a standard set of measures to make it possible to compare EDs or healthcare systems in a more uniform way. Although the framework was a helpful tool to compare EDs in different countries, we also showed that not all variables were available for each country limiting the possibility for comparison. Variables like number of nursing direct clinical care hours per 100 patient visits, would benefit from better definitions, because these variables are probably important for the quality of ED care. In addition, these variables are also important in order to be able to properly interpret the differences between countries. In the future, quality registries should register these variables in a reliable and consistent way.

Our study has several strengths, such as the large sample size in different countries and the use of the template for uniform reporting to compare countries. There are however also important limitations. First, this study has limitations inherent to the retrospective study design like the risk of information bias caused by human documentation errors and missing data. However, this risk is reduced by validating the data before using in the quality registers of the three contributing countries and the transfers of data are automatic. In addition, it was part of the feasibility aim of the study. Secondly, despite careful discussion and synchronization of variable definitions it cannot be ruled out that some variables have still a slightly different meaning in clinical practice. For example, in the Australian dataset 3 hospitals saw pediatric patients while 1 did not. In addition, it is possible that there are still some differences with respect to which patients are classified to certain categories of presenting complaints. Finally, it should be mentioned that some of the data are from 2016. It cannot be ruled out that in later years changes occurred in the participating EDs.

## Conclusion

Large differences exist across countries with respect to patient and ED characteristics and their outcomes across countries. Future studies with more in-depth analyses of these differences in in-hospital mortality and hospitalization are needed to find modifiable aspects of health care which could be used to improve quality of ED care. These in-depth analyses would only lead to meaningful conclusions if the measures of the recently developed template for uniform reporting, and more detailed information for case-mix corrections are registered.

## Supplementary Information

Supplementary file 1.

Supplementary file 2. 

## Data Availability

The data that support the findings of this study are available from the NEED foundation (www.stichting-need.nl) but restrictions apply to the availability of these data, which were used under license for the current study, and so are not publicly available. The Danish dataset is based on information from the Regional Patient Registration system, the Regional electronic patient file, the logistic system from the regional EDs, the laboratory and microbiological regional databases, the Danish National Patient register and the Danish Civil Registration system. Data are not publicly available. The data from Australia were collected from the “The Sydney Multicenter Emergency Department Sepsis Archive” with ethics approval received for secondary use of the data.A copy of the de-identified dataset was provided by eHealth NSW to The University of Sydney (Sydney, Australia, https://www.sydney.edu.au/) under a data sharing agreement where it is securely stored in the encrypted form. Data maybe requested by contacting these authorities. Data are however available from the authors upon reasonable request and appropriate permissions from the abovementioned authorities/foundations.

## Abbreviations

CCU: Coronary care unit
ED: Emergency department
DNR: Do not resuscitate
GDP: Gross domestic product
GP: General practitioner
ICU: Intensive care unit
IQR: Interquartile range
MCU: Medium care unit
NEED: Netherlands emergency department evaluation database
LOS: Length of stay
SD: Standard deviation

## Data availability

The data that support the findings of this study are available from the NEED foundation (www.stichting-need.nl) but restrictions apply to the availability of these data, which were used under license for the current study, and so are not publicly available. The Danish dataset is based on information from the Regional Patient Registration system, the Regional electronic patient file, the logistic system from the regional EDs, the laboratory and microbiological regional databases, the Danish National Patient register and the Danish Civil Registration system. Data are not publicly available. The data from Australia were collected from the “The Sydney Multicenter Emergency Department Sepsis Archive” with ethics approval received for secondary use of the data.A copy of the de-identified dataset was provided by eHealth NSW to The University of Sydney (Sydney, Australia, https://www.sydney.edu.au/) under a data sharing agreement where it is securely stored in the encrypted form. Data maybe requested by contacting these authorities.

Data are however available from the authors upon reasonable request and appropriate permissions from the abovementioned authorities/foundations.
