# Supplementary material for: Characteristics and outcomes of emergency department patients across health care systems: an international multicenter cohort study
Source: Int J Emerg Med. 2024 Sep 27;17:123. doi: 10.1186/s12245-024-00715-0 (PMC11437790; doi:10.1186/s12245-024-00715-0)
Supplement: Supplementary file 1 [file 12245_2024_715_MOESM1_ESM.docx]

**SUPPLEMENTARY FILES 1 and 2.**

**Supplementary file 1.** Study setting per country

*The Netherlands*

In the Netherlands, the population comprises approximately 18 million people who live in an area of 41.543 km². Patients are commonly referred to an ED by the GP after telephone contact and can be directly referred to a (super) specialist or an emergency physician. Besides that, approximately 30% of the patients come to the ED as a self-referral (13). If non-ambulant or in case of a life-threatening emergency, patients can be transported by ambulance. The emergency telephone number (112) can be called by patients or GPs. The mean time of ambulances from alarm time to arrival at the scene for very urgent and urgent calls are within 92% of the target times of 15 and 30 min, respectively. Four emergency helicopter services exist which are called depending on the nature of the health problem or trauma (9).

The included EDs in this study are staffed 24/7 by ED physicians, who supervise emergency medicine residents and/or physician assistants, and by residents of other specialties. The annual census differs across EDs but ranges from 15 to 55 thousand ED visits. Currently, there are ~80 EDs but the government is considering to reduce that number. There are seven tertiary care (academic) centres, the remaining being general or urban teaching hospitals. EDs also provide trauma care, and are classified as level 1, 2 or 3 trauma centres. Level 1 trauma centres have all resources, Level 2 only have neurosurgery or thoracic surgery, Level 3 have none of the above. 14.5% of the gross national product is spend on health care (14). For details and current criteria for EDs in the Netherlands see “kwaliteitskader spoedzorg” (15, 16).

*Denmark*

Denmark has 5.8 million inhabitants who live in an area of 42.933 km². The country consists of five independent health regions. Four of the five regions have GPs on call 24/7 and one region has a telephone service for prehospital emergency evaluation and treatment. In each region patients are referred to EDs by the GP, the telephone service, which can be called by patients or GPs, or they arrive by emergency ambulance after a 112 call. Apart from patients who arrive by 112 ambulance calls, only few come to the ED as a self-referral. The prehospital emergency medical system is three-tiered and consists of emergency medical technicians, paramedics, and prehospital physicians in ground- or helicopter-based emergency care units (10). There are 25 physician staffed ambulances and four helicopters in the country on duty 24/7 (17). The median time of ambulances between alarm time to arrival at the scene from very urgent calls is 8 minutes with interquartile range 6-11 minutes.

The EDs are staffed by ED consultants in a varying proportion of the day. The remaining part of the day the EDs are staffed by consultants from other specialties. The consultants supervise interns, residents, and other clinical staff. Annually, there are 1.8 million acute hospital contact of which most are provided at the EDs (18). Patients over 70 years of age comprise 22.6% of the acute hospital contacts. All acute care in Denmark is public and free of charge.

*Australia*

The population of Australia comprises of approximately 25.7 million people; of these 17 million live in the capital cities with the remaining population residing in rural and regional Australia (19). Patients can be referred to the ED by a GP, however 75% of patients present to the ED without referral. In case of emergency, either patient or a GP can call an ambulance, which can provide paramedic care, transport patients to the ED or transfer patients between hospitals. In 2018-2019, average response time ranged 14.7 – 21.3 minutes in capital cities and 14.9 – 29.2 minutes state-wide (20). There are 96 ambulance aircraft available nationally for trauma and other urgent patient transport. The EDs are staffed by ED consultants 24/7 who supervise interns, residents and other clinical staff. Annually, 8.8 million people present to the ED, patients over 65 years of age comprise the largest group of the ED presentations (22). Most of the emergency care is provided by public hospitals in Australia, there are 287 public hospital EDs in the country (21).

**Supplementary file 2.** Data collection per country

*The Netherlands*

For the Netherlands we collected data from the Netherlands Emergency department Evaluation Database (NEED), which is the Dutch quality registry for EDs. For this study data were available from four of the nine participating hospitals. The database contains one academic tertiary centre and three small to large urban hospital. The onset of participation differed per hospital, but all data were between the period 1 January 2017 until 1 September 2021.

The NEED stores data using the web-based application Project Manager Internet Server (ProMISe, Leiden, the Netherlands, https://www.msbi.nl/promise/promise.aspx). Privacy sensitive data are encrypted by a trusted third party (ZorgTTP, Houten, the Netherlands, www.zorgttp.nl) with Trusted Reversible Encryption Service.

*Denmark*

The database in Denmark was a research project accepted by Danish Patient Safety Authority. It contains information of all ED visits from patients above 18 years old in Region of Southern Denmark (population 1.2 million, served by five EDs) in the period 1 January 2016 until 19 March 2018. The dataset is based on information from the Regional Patient Registration system, the Regional electronic patient file, the logistic system from the regional EDs, the laboratory and microbiological regional databases, the Danish National Patient register and the Danish Civil Registration system. All data are linked by the Danish personal identification number and following linkage has been encrypted.

*Australia*

The data from Australia were collected from the “The Sydney Multicenter Emergency Department Sepsis Archive” with ethics approval received for secondary use of the data. A copy of the de-identified dataset was provided by eHealth NSW to The University of Sydney (Sydney, Australia, <https://www.sydney.edu.au/>) under a data sharing agreement where it is securely stored in the encrypted form. The data contain information of all ED visits from January 2017 to November 2019 of the four participating hospitals: Blacktown, Mt-Druitt, Westmead and Auburn. There was a total of 556.652 patients encounters in this period.
