# Supplementary material for: Characteristics and outcomes of emergency department patients across health care systems: an international multicenter cohort study
Source: Int J Emerg Med. 2024 Sep 27;17:123. doi: 10.1186/s12245-024-00715-0 (PMC11437790; doi:10.1186/s12245-024-00715-0)
Supplement: Supplementary file 2 [file 12245_2024_715_MOESM2_ESM.docx]

**SUPPLEMENTARY FILES 3-**

**Supplementary file 3.** Data registered in the NEED.

**GENERAL DATA OF PARTICIPATING EMERGENCY DEPARTMENTS**

In the NEED, several common details of the hospital and the emergency department are registered once. This data is updated every year. Every year a survey must be send to the head of the corresponding emergency departments, to obtain this information.

The NEED uses a web-based data management application Project Manager Internet Server (ProMISe, Leiden, the Netherlands) to store data, which is NEN7512 certified and complies with Good Research Practice requirements. Independent of ProMISe, data are encrypted by a trusted third party, i.e. the company ZorgTTP in Houten, the Netherlands.

All variables were prospectively registered in the digital hospital information system (Chipsoft, Amsterdam, Netherlands; Epic, Verona, USA) of each participating ED. Data were automatically transferred from the digital hospital information system to the NEED in ProMISe.

**1. Which hospital.**Coding: <Hospital Code>.

**2. Hospital status.**Urban=0. Urban(STZ=Foundation of top clinical hospitals)=1. Academic Medical Centre=2.

**3. Number of patients in the region of the relevant hospital.**This information can be obtained for each participating hospital by the NZa (Dutch Healthcare Authority; was once used to calculate the hospital budgets).

**4. Emergency physicians.**

1. Number of emergency physicians working at the emergency room in the hospital on the first of January of the year in question.
2. Emergency physicians in fulltime equivalent.
3. Number of residents in emergency medicine on the first of January of the year in question.
4. Number of residents not in training on the first of January of that year.

**5. Time presence of emergency physicians.**

1. Non-24/7 presence by emergency physicians=0. Minimum of 1 emergency physician 24 hours a day, 7 days a week=1.
2. How many emergency physicians, residents in emergency medicine and residents not in training are working average at the emergency department daily for patient care. Number per shift. The number of shifts for emergency physicians also must be defined.

**6. Number of trauma surgeons employed by the hospital (so NO general surgeons).**Trauma surgeons in fulltime equivalent.

**7. Number of internists in acute medicine employed by the hospital. (So NOT general internists).**

**8a. Trauma centre.**No=0. Level 2=1. Level 1=2.

**8b. Intensive care in hospital.**

Not present=0. Present=1.

**9. Medium Care (or high care) present in hospital.**Not present=0. Present=1.

**10. Number of visits per year on the emergency department (so NOT the number of emergency
patients per year).**This only accounts for the number of patients at the emergency department. If a separate emergency cardiac care is present or General Practice Center is integrated, these patient visits are counted separately.

**11. Number of emergency room patients per year.**N.

**12. General Practice Center integrated within the emergency department.**Not present=0. Present=1.

**13. Emergency cardiac care present AT or NEXT to the emergency department.**Not present=0. Present=1.

**14. Quality indicators by the NVSHA (Dutch professional association for Emergency Physicians).**See also the NVSHA website. www.nvsha.nl.

**Quality indicator for Acute Coronary Syndrome (ACS).**

**1. Are patients with (suspected) ACS treated at your emergency department?**Indicate whether any patients are suspected for acute coronary syndrome at your emergency department.
Answer: No=0. Yes=1.

**2. For patients with (suspected) ACS, do you have a protocol about administration of aspirin?**Indicate whether a protocol is used at the emergency department for the administration of aspirin in cases of suspected acute coronary syndrome. Mention which protocol. Answer: No=0. Yes=1.

**3. Time for administration of aspirin to patients with (suspected) ACS registered at the emergency room?**Specify whether the exact time of administration of aspirin given to patients with suspicion of acute coronary syndrome is registered at the emergency department. This can be a written or electronic registration. Answer: No=0. Yes=1.

**Quality indicator Complication registration.**A complication is an unintended and undesired event or condition during or following medical treatment which is harmful to the health of the patient needing change in medical treatment, or if irreversible health damage exists.

**1. Complication registrations.**Is a complication registration used at the emergency department?
Only one answer is possible. If this question is answered with 'no', the other questions with indicator 'Triage on Emergency Department' do not apply. You can continue to answer the questions with the next indicator. Answer: No=0. Yes=1.

**2. Complication meeting.**A complication meeting at least four times a year at the emergency room?
Answer only yes or no. Answer: No=0. Yes=1.

**3. Type of complication registration.**

Here we would like you to briefly specify which CR you are using. If possible, you can attach a copy of the list of complications registered. Please note: This does not concern completed lists with numbers of complications. *<Free text:…>.*

**Quality indicator child abuse/domestic violence.**

**1. Presence of child abuse protocols.**A written child abuse protocol used at the emergency department which meets the following minimum competencies?

1. A multidisciplinary child abuse team is active in the hospital.
2. Employees at the emergency department are trained in identifying child abuse.

Answer: Yes, to both=3. A yes, B no=2. A no, B yes=1. No=0.

**2. Screening instrument.**Does the ED use a screening instrument to signal child abuse, such as the SPUTOVAMO form or a derivative? Answer: No=0. Yes=1.

**Which screening instrument is used at the emergency room? (multiple answers possible):**SPUTOVAMO form. No=0. Yes=1.
Top - toe examination. No=0. Yes=1.
Otherwise, describe: *<Free text…>.*

**3. Percentage of completed screening documents.**What is the number of completed screening documents at the emergency department, in relation to the total number of children up to and including 18 years old at the emergency department?
n Number of completed screening documents at the ED in January 2011.
N Total number of children up to and including 18 years at the emergency room in January 2011.
n / N * 100% Screening document percentage.

**4. Protocol 'parent notifications'.**Is a protocol used at the emergency department for the reporting of children as victims of domestic violence / suicidal attempt / auto mutilation / excessive alcohol and / or drug use?
Answer: No=0. Yes=1. Comments: *<Free text…>.*

**Quality indicator pain relief.**

**1. Pain scoring systems.**Is a pain scoring system used at the emergency department?
Answer: No=0. Yes=1. If yes, which scoring system: *<Free text…>.*

**2. Time registration of pain relief.**Is the exact time recorded when pain relief is given in the emergency room?
Answer: No=0. Yes=1.

**3. Pain protocols.**

Is a pain protocol used at the emergency room?

Answer: No=0. Yes=1. Comments: *<Free text…>.*

**Quality indicator sepsis.**

**1. Sepsis protocol.**Is a sepsis protocol used at the emergency department?
Answer: No=0. Yes=1. If yes, which protocol: *<Free text…>.*

**2. Screening document.**Is a screening document used for sepsis in the emergency room?
Answer: No=0. Yes=1. If yes, which document: *<Free text…>.*

**3. Time registration for administering of antibiotics to patients with sepsis.**Is time of administering of antibiotics to patients with sepsis registered at the emergency department? Answer No=0. Yes=1. Comments: *<Free text…>.*

**Quality indicator Procedural Sedation and Analgesia (PSA).**

**PATIENT RECORDS.**The following basic principles are used:
1) Facts are recorded, e.g. time points. Derived variables are generated afterwards, such as length of stay.
2) Continuous variables are not categorized because this will lead to data loss.
3) Only create categories for coding, considering a remaining category 'other' and the possibility to indicate 'unknown'.

The following data are collected from all patients:

**Demographic information.**In general, the social service number (BSN) and date of birth are registered for each patient. In addition, a unique code is registered for each emergency room visit.

**ENCRYPTION BY ‘ZORG TTP’.**

**1. Age.**Age in years at the time of registration at the emergency registration desk.

**2. Gender.**Male or female. Code: Female=0. Male=1.

**3. Date and time registration of ED presentation.**DD-MM-YEAR-HOUR-MIN.

**Transport to the hospital.**

**4. Type of arrival at the hospital.**Arrival at the emergency room by ambulance or by own transport (i.e. walking, bicycle/moped or public transport). Coding: Own transport=0. Arrival with ambulance=1. Unknown=9.

**5. Referral status.**Self-referrer or referred by GP or another specialist. Coding: Self-referrer=0. Referred by physician=1. Referred by specialist=2. Unknown=9.

For variable 4 and 5, more variables are possible. This has to be discussed and agreed with the participating hospitals. Example: Anyone who enters by ambulance after a 112-call, would that be a self-referrer?

**General information on the ED.**

**6. Triage category.**Triage category according to Manchester Triage System upon arrival of patient. Coding: Blue=1. Green=2. Yellow=3. Orange=4. Red=5. If the Boston triage system is used, the corresponding categories 1 to 5 will be used. If the Netherlands Triage System is used, it will be entered.

**7. Main specialism or supervisor. In case this is not known, the specialty that admits the patient
will be recorded.**Coding: Emergency physician=0. Surgery=1. Internal medicine (or super specialism of internal medicine for example gastroenterologist or vascular medicine)=2. Cardiology=3. Neurology=4. Urology, otorhinolaryngology (Ear, Nose, Throat) or ophthalmology=5. Pediatrics=6. Other=7.

**8. Presenting complaint according to MTS triage algorithm.**So not the presenting complaint according to the registration desk employee. Coding: Complaint 1=1, Complaint 2=2, ........... Complaint 52=52.

**9. Shock room or trauma room.**Coding: Treatment in standard treatment room=0. Shock room/trauma room=1. The definition of a shock room needs to be determined by the participating emergency departments. They need to agree whether, for example, thrombolysis is part of this or not. The exact conditions of this room must be specified.

**10. Vital parameters at the time of triage.**So NOT mentioned on arrival at the treatment room. This variable only needs to be registered if it has been measured. If not measured, the space is left empty.
Oxygen saturation as measured with a pulse oximeter used in the hospital (percentages without oxygen). If measured when oxygen is given, note the number of liters / min of oxygen.
Systolic and diastolic blood pressure (mmHg).
Heart rate measured with pulse oximeter (beats / min.).
Temperature measured with an ear thermometer (degrees Celsius).
Glasgow coma scale (EMV score).

A separate variable is made of each vital parameter, with limit values and units. The Early Warning Score can then be calculated.

**11a. Additional blood tests on the emergency room.**If blood has not been obtained and sent for analyses, a 0 will be entered. If blood has been sent for analyses, a 1 will be entered.

**11b. If blood has been obtained, the following values are entered.**Carefully check whether the units at all EDs are the same!
Blood gas obtained. 0=No. 1=Yes. If yes: Venous=0. Arterial=1.
pH:…
PO2(KPa)
PCO2(KPa)
Bicarbonate(mmol/L)
B.E.:…

*Biochemistry:*Sodium(mmol / L)
K(mmol/L)

Creatinine(μmol / L)
Urea(mmol/L)
ASAT(U/L)
ALAT(U/L)
Y-GT(U/L)
AF(U/L)
LDH(U/L)
CK(U/L)
Highly sensitive Troponin(ng/L)
CRP(mg/L)
Pro-BNP(mg/L)
Procalcitonin(mg/L)
Lactate(mmol/L)

*Hematology:*Hb(mmol/L)
Leucocytes(x109/L)
Platelets(x1012/L)
D-dimer(mg/L)
INR

*Toxicology:*Ethanol(mg/L)
Paracetamol(mg/L)

*Blood cultures collected at the emergency room and sent for cultures:*No=0. Yes=1. Blood cultures should be at least one set (i.e. aerobic and anaerobic).

*Urine collected at the emergency room and send for cultures:*No=0. Yes=1.

*Urinary sediment collected at the emergency room:*No=0. Yes=1.

*Urine toxicological analysis performed at the emergency department:*No=0. Yes=1.

**12a. Additional X-ray diagnostics at the emergency room.**If no X-ray diagnostics have been performed at the emergency room, a 0 is entered. If a type of X-ray diagnostics has been performed at the emergency room, a 1 is entered. Definition: 0 should also be entered if the patient is sent for an X-ray by the GP, and afterwards sent to the emergency department.

**12b. If X-ray diagnostics have been performed, the type of examination must be recorded.**Conventional X-ray, extremity. No=0. Yes=1.
Conventional X-ray, divers (including chest X-ray). No=0. Yes=1.
Abdominal ultrasound (including FAST). No=0. Yes=1.
Ultrasound for deep vein thrombosis (DVT). No=0. Yes=1.
Ultrasound, divers. No=0. Yes=1.
Head CT. No=0. Yes=1.
CT-cervical spine. No=0. Yes=1.
CT-pulmonary embolism. No=0. Yes=1.
CT-aorta. No=0. Yes=1.
CT-chest-abdomen (trauma). No=0. Yes=1.
Patients can receive multiple examinations at the emergency room.

**13. Number of consultations at the emergency room.**Coding: No consultations=0. One consultation=1. Two consultations=2. Three consultations=3, etc.

**Outcomes**.

**14. Duration of stay at the emergency room.**Duration in minutes. Discharge time of the emergency room minus the registration time at the emergency room. (So NOT the announcement or triage time).

**15. Discharge destination (disposition).**Discharged home (NH), to standard department (AFD), medium care unit (MCU), coronary care unit (CCU) or intensive care unit (ICU). Died in the emergency room. Transfer to another hospital. Scheduled outpatient check-up after emergency room visit and related to emergency room visit. Coding: NH=0, AFD=1, MCU or CCU=2. ICU=3. Deceased at SEH=4. Transfer=5. Scheduled outpatient check-up=6. Referral to General Practice Center=7.

**16. Hospital length of stay.**Hospital length of stay in days. Discharge date minus recording date. If a patient at the emergency room is discharged home, the hospital length of stay is 0 days. If people are registered before midnight and leave the ED after midnight, this should be accounted as 0 days!

**17. In-hospital mortality.**Coding: Leaving the hospital alive=0. Died in emergency room or brought in dead (for example during CPR)=1. Died in hospital before discharge=2.

**18. Return to ED with medical problem/complaints.**Coding: No return with medical problems within 7 days after registration time on ED=0. Return to the ED within a week after discharge with a medical problem which may or may not be related to the previous ED visit=1. Return to the ED within a week after discharge with a medical problem which is clearly related to the previous ED visit=2. Otherwise=3.

**19. ICD-10 code and diagnosis after discharge from the emergency department and hospital.**

**20. Diagnosis Treatment Code (DBC or DOT).**Determine whether these are the same in every hospital.

**Supplementary file 4.** Synchronization presenting complaints

Supplementary file 4.1. Synchronization of MTS and NTS presenting complaints of the NEED
The participating EDs in the NEED use different triage systems for registration of the presenting complaints. The Netherlands Triage system (NTS) includes 50 presenting complaints and the Manchester Triage System (MTS) includes 51 presenting complaints. We merged the NTS and MTS presenting complaints into one combined list as shown below.

| **Synchronized presenting complaints used in this study** | **MTS presenting complaints** | **NTS presenting complaints** |
| --- | --- | --- |
| Abdominal pain | Abdominal pain child  Abdominal pain adult | Abdominal pain child  Abdominal pain adult |
| Abscesses and local infections | Abscesses and local infections | - |
| Allergy & insect bite | Allergy | Allergy or insect bite |
| Apparently drunk | Apparently drunk | - |
| Assault | Assault | - |
| Asthma | Asthma | - |
| Back pain | Back pain | Back pain |
| Behaving strangely or suicidal | Behaving strangely | Behaving strangely or suicidal |
| Breast infection |  | Breast infection |
| Burns & scalds | Burns & scalds | Burns & scalds |
| Chest pain | Chest pain | Chest pain |
| Collapse | Collapsed adult | Dizziness  Collapse |
| Constipation |  | Constipation |
| Coughing |  | Coughing |
| Crying baby | Crying baby | - |
| Dental problems | Dental problems | Dental problems |
| Diabetes | Diabetes | Diabetes |
| Dyspnea | Shortness of breath in child  Shortness of breath in adults | Shortness of breath |
| Ear problems | Ear problems | Ear problems |
| Exposure to chemicals | Exposure to chemicals | - |
| Extremity problems | Extremity problems | Arm problems  Leg problems  General/extremity trauma |
| Eye problems | Eye problems | Eye problems |
| Facial problems | Facial problems | Nosebleed  Facial trauma |
| Feeling unwell | Unwell/sick child  Unwell/sick adult | Unwell adult  Sick child  Fever in child  Fever in adult  Neurological failure |
| Foreign body | Foreign body | Foreign body |
| Gastro-intestinal bleeding | Gastro-intestinal bleeding | - |
| Genital problems | Testicular pain | Genital problems |
| Headache | Headache | - |
| Implantable Cardioverter Defibrillator (ICD) |  | Implantable Cardioverter Defibrillator (ICD) |
| Irritable child | Irritable child | - |
| Limping child | Limping child | - |
| Major incidents primary | Major incidents primary | - |
| Mental illness | Mental illness | - |
| Near-drowning |  | Near-drowning |
| Neck pain | Neck pain | Neck problems  Neck trauma |
| Overdose & poisoning | Overdose & poisoning | Poisoning |
| Palpitations | Palpitations | Palpitations |
| Per vaginum bleeding | Per vaginum bleeding | Per vaginum bleeding |
| Pregnancy | Pregnancy | Childbirth |
| Rashes | Rashes | Rashes |
| Rectal problems |  | Rectal problems |
| Seizure | Seizure | Seizure |
| Self-harm | Self-harm | - |
| Sexually acquired infection | Sexually acquired infection | - |
| Throat problems | Sore throat | Throat problems |
| Trauma | Trauma head  Trauma torso  Major trauma  Falls | Abdominal trauma  Back trauma  Head trauma  Thorax trauma |
| Urinary problems | Urinary problems | Urinary problems |
| Vomiting & diarrhea | Vomiting & diarrhea | Vomiting  Diarrhea |
| Wounds | Wounds | Wounds |

Supplementary file 4.2. Synchronization of top 18 presenting complaints of the three countries
The participating countries use different triage systems and have different presenting complaints in their top 18. We merged the top 18 presenting complaints of the three participating countries as much as possible into a combined list as shown below.

| **The Netherlands** | **Denmark** | **Australia** | **Merge** |
| --- | --- | --- | --- |
| Extremity problems | Extremity problems | Pain limb | Extremity problems |
| Feeling unwell | Feeling unwell or delier  Fever  Neurological | Unwell  Fever | Feeling unwell |
| Abdominal pain | Abdominal pain | Pain abdominal | Abdominal pain |
| Dyspnea | Respiratory problems inclusive dyspnea | Respiratory - shortness of breath | Dyspnea |
| Chest pain | Chest pain | Pain chest | Chest pain |
| Trauma | Trauma | Injury/trauma  Falls | Trauma |
| - | Minor trauma | - | Minor trauma |
| Wounds | Wounds | - | Wounds |
| Collapse | Collapse | Dizziness | Collapse |
| Palpitations | Palpitations | - | Palpitations |
| Urinary problems | - | - | Urinary problems |
| Headache | Headache | Headache | Headache |
| Overdose & poisoning | Overdose & poisoning | - | Overdose & poisoning |
| Facial problems | - | - | Facial problems |
| Diarrhea & vomiting | Diarrhea & vomiting | Vomiting/diarrhea | Diarrhea & vomiting |
| Eye problems | - | Eye problems | Eye problems |
| Abscesses & local infections | Abscesses & local infections | Abscess/local infection | Abscesses & local infections |
| Behaving strangely of suicidal | - | - | Behaving strangely of suicidal |
| Back pain | Back pain | Back pain | Back pain |
| - | - | Care – patient review | Care – patient review |
| - | - | Mental health | Mental health |
| - | - | Coughing | Coughing |
| - | - | Bleed per vaginum | Bleed per vaginum |
| - | LAB abnormalities | - | LAB abnormalities |
| - | GI bleeding | - | GI bleeding |
| Others | Others | Others | Others |

**Supplementary file 5.** Sample size calculation Danish and Australian registries

The Danish registry currently contains 408.673 ED patients. To prevent overfitting, we use the rule of thumb that approximately 10 events per potential predictor are needed. The estimated frequency of in-hospital mortality is ~2% and the estimated hospitalization rate is ~40%. This comes down to 8173 in-hospital deaths and 163469 hospitalized patients. A maximum of 817 potential confounders can therefore be included in the model for case-mix adjusted in-hospital mortality and 16347 potential confounders for case-mix adjusted hospitalization. The number of potentially independent determinants in our models will not exceed these numbers.

The Australian database contains 556.652 ED patients. To prevent overfitting, we use the rule of thumb that approximately 10 events per potential predictor are needed. The estimated frequency of in-hospital mortality is ~4% and the estimated hospitalization rate is ~29%. This comes down to 22266 in-hospital deaths and 161429 hospitalized patients. A maximum of 2227 potential confounders can therefore be included in the model for case-mix adjusted in-hospital mortality and 16143 potential confounders for case-mix adjusted hospitalization. The number of potentially independent determinants in our models will not exceed these numbers.

**Supplementary file 6.** Definitions of variables used in Table 1

| Variable collected | Short description |
| --- | --- |
| Age | Age in years of time at registration at the ED desk |
| Sex | Male or female, registration at the ED desk |
| Triage category | Triage category according to MTS, NTS or ATS.   - Non-urgent & standard: MTS or NTS blue & green, ATS 4 &5. Low urgency. For example twisted ankle, wound, contusion, insect bite. - Urgent: MTS and NTS yellow, ATS 3. For example sepsis, pneumonia, chest pain, dyspnea - Very urgent: MTS and NTS orange, ATS 2. For example sepsis, pneumonia, chest pain, dyspnea - Immediate: MTS or NTS red, ATS 1. Immediate care required. For example shock, coma, advanced life support |
| Top 18 presenting complaints | For the Netherlands, we merged the MTS and NTS presenting complaints and looked at the top 18 complaints which are most often registered at the ED (see supplementary file 4.1). Besides that we also merged the top 18 presenting complaints per country as much as possible (see supplementary file 4.2) |
| Charlson Comorbidity Index | A tool to measure the amount of comorbidity for patients with specific comorbid conditions. This is used to predict outcomes like hospitalization and mortality. Higher scores imply a more severe condition. |
| Registered specialty | These numbers are based on how the patients are registered, so it is not a reflection of who is in reality the treating doctor.  The treating specialty includes: ED physician, Medicine (internal medicine, pulmonary medicine, cardiology, neurology), Surgery (general -, trauma -, orthopedic surgery, urology, ophthalmology, ENT (ear, nose, throat), Pediatrics and Others. |
| Arrival mode | Whether patients arrived by own transport or by ground or air ambulance. |
| Vital signs | This contains the vital signs measured at time of triage, before ED treatment. |
| Blood tests | Whether blood has obtained for testing. This includes various forms of biochemistry, hematology and toxicology. |
| Radiological imaging | Whether any form of radiological imaging was done. This contains X-ray, ultrasound and CT-scan. |

**Supplementary file 7.** Definitions of variables used in Table 2 according to the Utstein framework

| Variable collected | Short description |
| --- | --- |
| Number of GPs / 1000 inhabitants | The total number of general practitioners in the country divided by the amount of inhabitants in that country * 1000  * Source the Netherlands: de Staat van Volksgezondheid en Zorg. <https://www.staatvenz.nl/kerncijfers/huisartsen-aantal-werkzaam> 🡪12766 GPs in the Netherlands with 17.34 million inhabitants in 2019.  * Source Australia: Australian Government. Department of Health. <https://www.health.gov.au/health-topics/doctors-and-specialists/in-australia> 🡪 31000 GPs in the Netherlands with 25.69 million inhabitants in 2020. |
| Number of ambulance staffing / 1000 inhabitants | The total number of ambulance staffing in the country divided by the amount of inhabitants in that country * 1000  * Source the Netherlands: Ambulancezorg Nederland. <https://www.ambulancezorg.nl/sectorkompas/facts-figures-2018-copy-copy> 🡪 6541 ambulance staffing in the Netherlands with 17.44 million inhabitants in 2020.  * Source Australia: Australian Government Productivity Commission 2018-19 <https://www.pc.gov.au/research/ongoing/report-on-government-services/2021/health/ambulance-services> |
| Target time to patient | The obligatory target time for ambulance care to arrive at patients after receiving a very urgent call. |
| Number of acute care beds/ 1000 inhabitants | The number of beds on ICU, surgical units, gynecological and obstetric services and acute psychiatric care beds in the whole country (not only in the hospital included in the database) divided by the amount of inhabitants in that country * 1000  * Source Australia and the Netherlands: OECD (Organization for Economic Co-operation and Development) <https://www.oecd.org/coronavirus/en/data-insights/hospital-beds-acute-care> |
| Number of hospital beds / 1000 inhabitants | Total number of hospital beds regularly occupied and immediately available for the care of inpatients. Curative care beds, rehabilitation care beds, long-term care beds and other hospital beds were included.  * Source the Netherlands (national): Staat van Volksgezondheid en Zorg. <https://www.staatvenz.nl/kerncijfers/ziekenhuisbedden> 🡪 39900 hospital beds in the Netherlands with 17.34 million inhabitants in 2019.  * Source Australia: Australian Government Productivity Commission 2018-19  [https://www.pc.gov.au/research/ongoing/report-on-government-services/2021/health/public-hospitals](https://www.pc.gov.au/research/ongoing/report-on-government-services/2021/health/public-hospitals 4) |
| Number of persons in adherence areas | Number of persons in the adherence area of a particular hospital divided by the amount of hospitals in that area. |
| Number of treatment spaces | The number of rooms designed for patient-centered care for an acute clinical problem and the resuscitation care spaces at the ED. Rooms where patients only were for a specific procedure or rooms for single-organ examination were not included. |
| Number of resuscitation spaces | The number of specific rooms at the ED used for high urgency patients or trauma care. These rooms contain a lot of equipment to act quickly. |
| Number of short stay unit spaces | The number of spaces at the unit for short-term treatment. These units may be separated from the ED. The spaces used when there was ED overflow or spaces which were managed by in-hospital teams were not included. |
| Number of direct clinical care hours by physicians per 100 ED visits | The number of emergency physicians or - residents and the amount of hours per week they work added to the estimated amount of hours physicians or residents from another specialism are present at the ED per week. That total amount divided by the number of ED visits in a week * 100. |
| Number of direct clinical care hours by nurses per 100 ED visits | The number of emergency nurses and the amount of hours per week they work. That total amount divided by the number of ED visits in a week * 100 |
| Emergency medicine specialist in the ED 24/7 | Whether there was at least one ED specialist at the ED 24/7 in all contributing hospitals in the database. |
| Total ED census | The number of ED visits per year in one country.  * Source the Netherlands: Nederlandse Zorgautoriteit (NZA). <https://puc.overheid.nl/nza/doc/PUC_301126_22/1/#:~:text=Tussen%202016%20en%202019%20sloten,de%20dichtstbijzijnde%20seh%20worden%20gebracht>. |
| Length of ED stay | Length of stay at the emergency department in hours for all patients who visit the ED |
| Length of ED stay for patients discharged home | Length of stay at the emergency department in hours for the patients who were discharged home after their visit. |
| 7-day ED revisit | Whether patients returned within a week after discharge with a medical problem which was related to or unrelated to the previous ED visit |

| Variable collected | Short description |
| --- | --- |
| Length of ED stay for all patients | Length of stay at the emergency department in hours for all patients who visited the ED |
| Length of ED stay for patients discharged home | Length of stay at the emergency department in hours for the patients who were discharged home after their visit. |
| Hospital admission directly after ED stay | Hospital admission includes admission to a normal ward, CDU, MCU, CCU, ICU or admission after transport to another hospital. |
| Length of hospital stay | The amount of days patients were admitted to a ward or specialized care unit in the hospital. If patients were administered to the hospital for less than 24 hours, this counts as 0 days. |
| In-hospital mortality | The number of patients who died at the emergency department, patients who were brought in dead or patients who died in the hospital before discharge. |
| 30-day mortality | The amount of patients died 30 days after their ED visit |
| 7-day ED revisit | Whether patients returned within a week after discharge with a medical problem which was related to or unrelated to the previous ED visit |

**Supplementary file 8.** Definitions of variables used in Table 2
